# Remote ischemic preconditioning differentially attenuates post-ischemic cardiac arrhythmia in streptozotocin-induced diabetic versus nondiabetic rats

**DOI:** 10.1186/s12933-017-0537-3

**Published:** 2017-04-26

**Authors:** Zhaoyang Hu, Mou Chen, Ping Zhang, Jin Liu, Geoffrey W. Abbott

**Affiliations:** 1 Laboratory of Anesthesiology & Critical Care Medicine, Translational Neuroscience Center, West China Hospital, Sichuan University, Chengdu, Sichuan China; 2 Bioelectricity Laboratory, Dept. of Pharmacology and Dept. of Physiology and Biophysics, School of Medicine, University of California, Irvine, CA USA

**Keywords:** Remote liver ischemic preconditioning, Diabetes, Arrhythmia, RISK pathway, Cardioprotection

## Abstract

**Background:**

Sudden cardiac death (SCD), a leading cause of global mortality, most commonly arises from a substrate of cardiac ischemia, but requires an additional trigger. Diabetes mellitus (DM) predisposes to SCD even after adjusting for other DM-linked cardiovascular pathology such as coronary artery disease. We previously showed that remote liver ischemia preconditioning (RLIPC) is highly protective against cardiac ischemia reperfusion injury (IRI) linked ventricular arrhythmias and myocardial infarction, via induction of the cardioprotective RISK pathway, and specifically, inhibitory phosphorylation of GSK-3β (Ser 9).

**Methods:**

We evaluated the impact of acute streptozotocin-induced DM on coronary artery ligation IRI-linked ventricular arrhythmogenesis and RLIPC therapy in rats.

**Results:**

Post-IRI arrhythmia induction was similar in nondiabetic and DM rats, but, unexpectedly, DM rats exhibited lower incidence of SCD during reperfusion (41 vs. 100%), suggesting uncontrolled hyperglycemia does not acutely predispose to SCD. RLIPC was highly effective in both nondiabetic and DM rats at reducing incidence and duration of, and increasing latency to, all classes of ventricular tachyarrhythmias. In contrast, atrioventricular block (AVB) was highly responsive to RLIPC in nondiabetic rats (incidence reduced from 72 to 18%) but unresponsive in DM rats. RISK pathway induction was similar in nondiabetic and DM rats, thus not explaining the DM-specific resistance of AVB to therapy.

**Conclusions:**

Our findings uncover important acute DM-specific differences in responsiveness to remote preconditioning for ventricular tachyarrhythmias versus AVB, which may have clinical significance given that AVB is a malignant arrhythmia twofold more common in human diabetics than nondiabetics, and correlated to plasma glucose levels >10 mmol/L.

## Background

Sudden cardiac death (SCD), in which failure of cardiac electrical activity leads to lethal cessation of the heartbeat, kills >1 in 1000 people annually in developed countries [1]. SCD is thought to require an external trigger, and also a substrate such as ischemia or heart failure, even though it typically presents in the absence of evidence of a prior myocardial infarct. Especially in younger victims, an ischemic substrate may be lacking, and in these cases an electrical substrate stemming from inherited disruption of one or more genes encoding ion channels, or the proteins that regulate them, is often implicated [2].

Human diabetes mellitus (DM) is an established risk factor for both myocardial ischemia and for SCD [3–5]. Hyperglycemia increases the risk of coronary artery disease, a predisposing factor for SCD [6], although in Type 2 DM it is difficult to separate hyperglycemia from insulin resistance, which in itself predisposes to coronary artery disease [7, 8]. In addition, DM also increases the risk of both SCD and non-sudden cardiac death (NSCD) independent of both known baseline cardiac disease, and of occurrence of non-fatal cardiac events. Strikingly, even prediabetic impaired glucose tolerance is associated with increased risk of SCD, NSCD and non-fatal cardiac events [9]. The link between DM and SCD may also contribute to ethnic disparity in the prevalence of SCD. Thus, incidence of sudden cardiac arrest (SCA) in blacks was recently found to be twice that of whites in a study based in Oregon, United States; in the same analysis, blacks had a higher prearrest prevalence of DM (52 vs. 33% in whites) but there was no difference in incidence of prearrest coronary artery disease or ventricular dysfunction (although interestingly, the baseline QT_c_ interval was 13 ms longer in blacks than whites) [10].

DM and even prediabetes can alter cardiac electrical activity, and this may contribute to the link between DM and SCD independent of coronary artery disease. Recently, it was found that in a prediabetic rat model, a high fructose-fat diet increased serum triglycerides as expected, but also slowed cardiac conduction velocity and prolonged the body-surface ECG QRS complex, without causing cardiac fibrosis or altering gap junctional coupling, Na^+^ or K^+^ currents. Importantly, the prediabetic rats were also more prone to ischemia/reperfusion injury (IRI) induced ventricular fibrillation than their non-prediabetic counterparts [11], reminiscent of the association between prediabetes and increased risk of SCD and other cardiac events observed in human populations [9].

We and others previously showed that remote ischemic preconditioning, in which ischemia is induced in extracardiac tissues, is cardioprotective in the context of subsequent cardiac IRI. Specifically, remote liver ischemic preconditioning (RLIPC) protected against SCD, and reduced infarct size, in two different IRI protocols performed in rats [12, 13]. In both cases, inhibition of cardiac glycogen synthase kinase-3β (GSK-3β) via phosphorylation was strongly implicated as a primary mechanism of RLIPC-induced cardioprotection. Here, given the aforementioned links between DM and SCD, and the need to understand mechanisms of both pathogenesis and cardioprotection in the context of DM-linked SCD, we examined the effects of acute streptozotocin-induced DM on IRI-triggered ventricular arrhythmias and SCD, and the therapeutic efficacy of RLIPC in diabetic versus nondiabetic rats.

## Methods

### Animals

The experimental procedures and protocols were approved by the Institutional Animal Care and Use Committee of Sichuan University Sichuan, China, (approval number: 2015035A). All rats were treated in accordance with the recommendations in the Guide for the Care and Use of Laboratory Animals of the National Institutes of Health (NIH Publication 8th edition, 2011). Male Sprague–Dawley rats (250–300 g, 7–8 weeks of age) were purchased from Chengdu Dashuo Experimental Animal Research Center (Chengdu, China). Rats were housed in a conditioned environment, i.e. 12-h light–dark cycle, 20–25 °C, 60 ± 5% relative humidity, free access to food and water.

### Induction of type 1 diabetes mellitus

Acute Type 1 DM was induced by a single intraperitoneal injection of streptozotocin (STZ, 50 mg/kg, Sigma Aldrich, USA) dissolved in 0.1 M citrate buffer (pH 4.5). Non-STZ groups received 0.1 M citrate buffer alone. Blood glucose levels were measured 1 week after STZ injection using a One Touch Ultra Glucose meter (Roche, USA). Rats with glucose levels ≥20 mmol/L were classified as diabetic, and those with blood glucose levels ≤20 mmol/L were excluded from the experiment.

### Surgical procedure

**Fig. 1:**
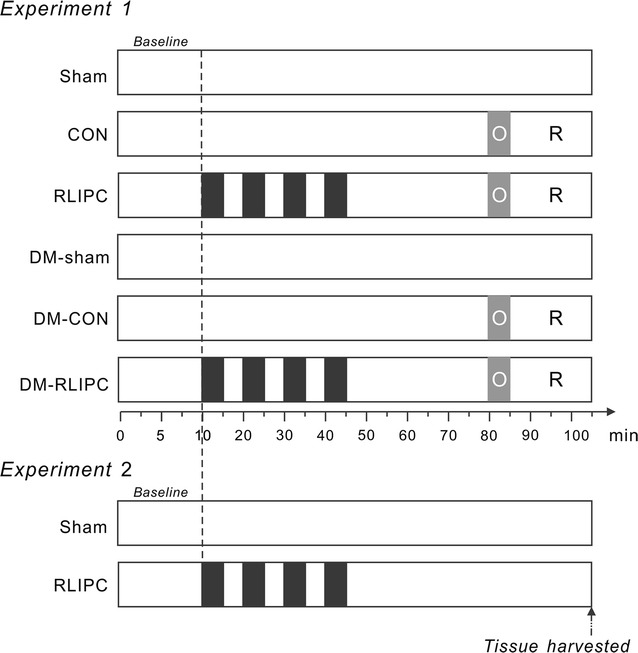
Experimental time-courses. Time courses for protocols in Experiments 1 and 2. *Black* liver ischemia cycles; *gray* with *white* O, left main coronary artery occlusion; *R* cardiac reperfusion, *CON* control, *RLIPC* remote liver ischemia preconditioning, *DM* STZ-induced diabetes

Rats were weighed before the experiments. Heart surgery was performed following the methods of our previous studies [12, 14, 15], with minor modifications (Fig. 1). In brief, rats were anesthetized with sodium pentobarbital (50 mg/kg, i.p.). The adequacy of anesthesia was controlled by monitoring the lack of response to toe-pinching, the absence of corneal reflex, and no response to surgical manipulation. Rats were artificially ventilated throughout the experiments. To maintain anesthesia, additional sodium pentobarbital (20 mg/kg) was administered every 30 min. All rats were subjected to 10 min stabilization after surgical preparation and instrumentation. The left chest of each rat was opened to identify the main left coronary artery, which was tied with a 6-0 silk ligature (Ethicon, Somerville, NJ, USA). Five minutes after ligation, the suture was released and then 20 min of reperfusion was performed. In the ischemic zone, a valid coronary artery occlusion was confirmed by the presence of regional dyskinesia and epicardial cyanosis. Reperfusion was confirmed by visualizing an epicardial hyperemic response. Cardiac activity was continuously recorded using a standard limb lead II configuration electrocardiographic system (Powerlab/8sp system, AD Instruments, Colorado Springs, CO, USA) throughout the experiment. RLIPC was achieved by clamping the portal vein, hepatic arterial and venous trunk for four cycles of 5 min of liver ischemia with 5 min intermittent reperfusions (liver I/R cycles).

### Experimental protocol

The experimental protocol for Experiment 1 is shown in Fig. 1. Rats were randomly assigned to the following groups:

1. Nondiabetic sham group (sham): the hepatic arterial and venous trunk, together with the portal vein were isolated. Chests were opened and coronary artery was exposed. The suture placed under the vessel bed was not tightened.
2. Nondiabetic control group (CON): rats were administered a 5-min left main coronary artery ligation followed by 20 min reperfusion. No hepatic intervention was performed.
3. Nondiabetic with remote liver ischemic preconditioning treatment (RLIPC): rats were subjected to four cycles of 5 min of hepatic ischemia and 5 min of reperfusion before myocardial ischemia. Hepatic ischemia was induced by clamping the vessel with a microvascular clip (ischemia period), and reperfusion was initiated by releasing the clip (reperfusion period).
4. STZ-induced diabetic sham group (DM-sham): STZ was injected to induced diabetes, chests and livers were exposed without further occlusion.
5. STZ-induced diabetic control group (DM-CON): diabetic rats were subjected to 5-min left main coronary artery ligation followed by 20 min reperfusion.
6. STZ-induced diabetic rats with remote liver ischemic preconditioning treatment group (DM-RLIPC): diabetic rats were subjected to hepatic I/R stimuli ahead of myocardial ischemia.

At the end of each reperfusion period, rats were euthanized with an overdose of sodium pentobarbital (200 mg/kg, i.p.). Death was confirmed by observing lack of respiration and cardiac activity. Evans blue (Sigma, St. Louis, MO, USA) at 1% was then administrated into the left ventricular cavity to depict the myocardial ischemic area at risk (AAR). The AARs were separated and weighed. AAR size (weight) was expressed as a percentage of the left ventricular weight. The tissue constituting the AAR region was then stored in a −80 °C freezer for subsequent protein phosphorylation analysis. In a parallel experiment (Experiment 2), to exclude the possibility that the liver ischemic stimuli might lead to hepatic damage, without further myocardial manipulation, liver I/R stimuli were conducted and tissue samples were taken at the same time points as those in Experiment 1.

### Arrhythmia analysis

Arrhythmia was measured as we described previously [12, 14, 16]. Briefly, parameters included: (1) Number of rats exhibiting cardiac arrhythmia; (2) SCD during the 20 min reperfusion period; (3) ventricular tachycardia (VT) (4) polymorphic VT (PVT); (5) ventricular fibrillation (VF); (6) AV block (AVB); in addition we recorded (7) the onset time of the first run of VT or VF, and (8) VT duration.

### Blood serum biochemistry

After the experiments, blood samples were collected and centrifuged at 2000 RCF for 10 min at 4 °C, then were frozen at −20 °C until analysis. Serum levels of aspartate aminotransferase (AST), alanine aminotransferase (ALT), lactate dehydrogenase (LDH), creatine kinase (CK) and α-hydroxybutyrate dehydrogenase (α-HBDH) were measured automatically using a BS-120 automatic biochemical analyzer (Mindray, Shenzhen, China).

### Liver studies

A separate group of experimental rats (Experiment 2), parallel to that used for arrhythmia analysis, was used for detecting possible hepatic damage upon RLIPC treatment. Without cardiac intervention, after liver I/R stimuli, liver histology examinations were performed. The liver sections were dissected at room temperature and immersed in 10% phosphate-buffered formalin solution overnight. After embedding in paraffin, liver slices (5 mm thick) were mounted on glass slides for haematoxylin and eosin (H&E) staining.

### Western blotting

Hearts were homogenized in lysis buffer containing 50 mM Tris–HCl pH 7.4, 150 mM NaCl, 1% NP-40, 1 mM EDTA, 0.25% sodium deoxycholate, phosphatase inhibitor cocktail (Sigma-Aldrich, St. Louis, MO, USA), and a protease inhibitor cocktail (Sigma-Aldrich, St. Louis, MO, USA). Homogenates were next centrifuged for 10 min at 10,000*g* at 4 °C. Protein concentration of the supernatant was determined by the BCA method (Pierce, Rockford, IL, USA), and after normalization for total protein concentration, samples were heated at 95 °C, loaded onto 12% SDS-PAGE gels (15 μg/well), separated by electrophoresis and then transferred onto nitrocellulose membranes (VWR, Batavia, IL, USA). Membranes were blocked at room temperature for 1 h and then incubated overnight at 4 °C with primary antibodies raised against phosphorylated extracellular signal-regulated kinase1/2 (ERK1/2) (Thr202/Tyr204), total ERK1/2, phosphorylated glycogen synthase kinase-3β(Ser9) (p-GSK-3β Ser9), total -GSK-3β, phospho-Akt (Ser473, p-Akt), total Akt, (all: rabbit, 1:1000, from Cell Signaling Technology, Danvers, MA, USA). Horseradish peroxidase (HRP)-conjugated goat anti-rabbit IgG was used as secondary antibody for detection using enhanced chemiluminescence (Millipore, Billerica, MA, USA). Bands were visualized using an Amersham Imager 600 system (GE healthcare, Little Chalfont, UK). Band densities were analyzed with ImageJ Data Acquisition Software (National Institutes of Health, Bethesda, MD, USA) and expressed as percent density compared to same-gel sham or control sample band densities.

### Statistical analysis

All values are expressed as mean ± standard error of mean (SEM). Unpaired two-tailed student’s t tests were used for comparisons between two values. Fisher’s exact test was employed to compare numbers of rats falling into one of two groups. Homogeneity of variance was tested by Levene’s test. For multiple comparisons between groups of three or more, one-way ANOVA followed by Newman-Keuls test was used if variances were equal; otherwise, Dunnett’s T3 test was utilized. Values of P less than 0.05 were considered statistically significant. All tests were two-sided.

## Results

### Liver I/R stimulus does not affect liver function

**Fig. 2:**
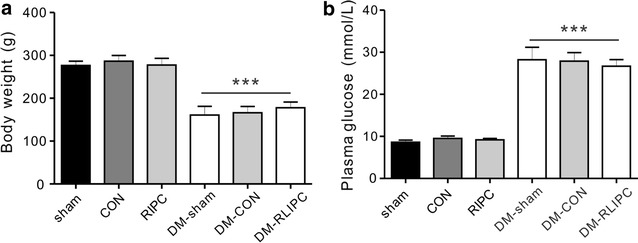
Effects of STZ treatment on body weight and plasma glucose. **a** Mean body weight and **b** baseline blood glucose concentrations of nondiabetic or diabetic rats with or without RLIPC treatment (*n* = 6–9). *CON* control, *RLIPC* remote liver ischemia preconditioning, *DM* STZ-induced diabetes; ***P < 0.001 vs. nondiabetic rats

**Fig. 3:**
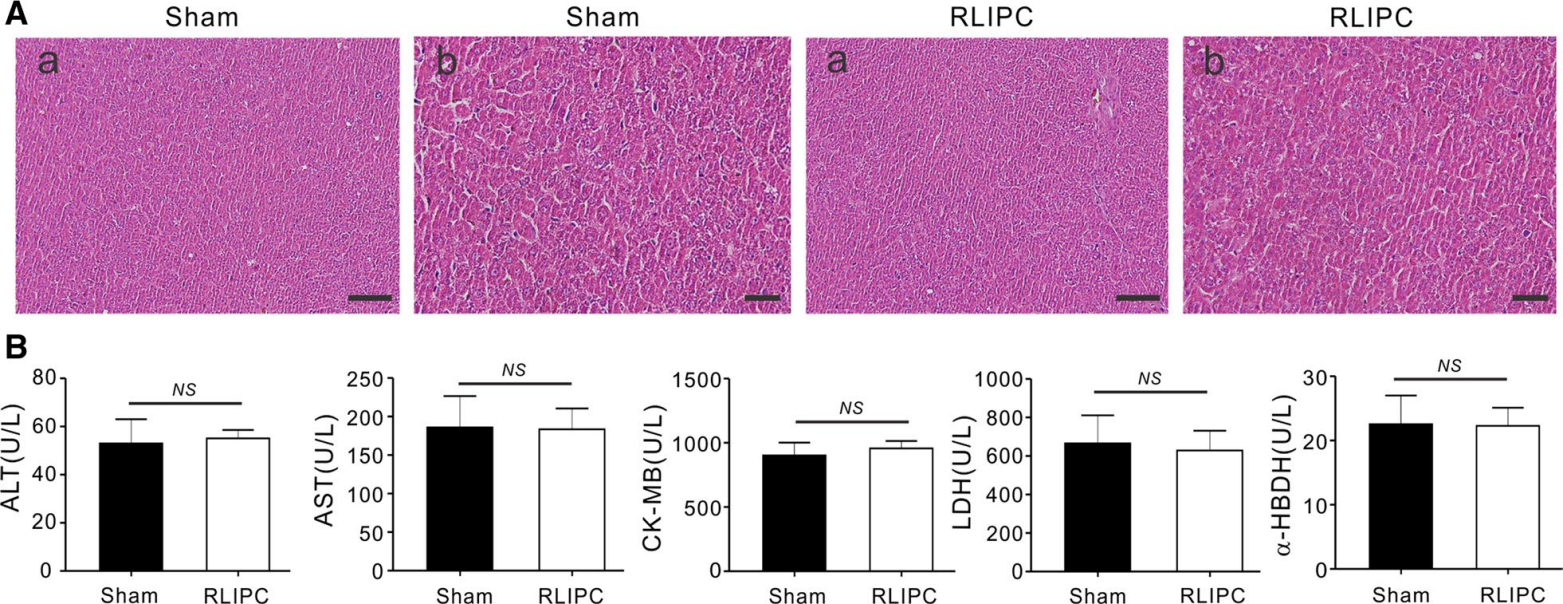
Liver preconditioning did not cause hepatic damage. **A** Representative (of *n* = 5 rats/group) H&E-stained liver sections of sham and RLIPC rats. *Scale bars* (*a*) 100 μm; (*b*) 10 μm. **B** Mean serum levels of AST, ALT, CK-MB, LDH, and α-HBDH in sham and RLIPC rats. *NS*, P > 0.05 among groups

After 7 days, the body weight of STZ-treated rats was decreased by more than one-third (P < 0.0001 vs. non-STZ treated rats, Fig. 2a). STZ-induced diabetic rats displayed hyperglycemia, evidenced as significantly increased plasma glucose, compared with age-matched non-STZ treated ones (all P < 0.001, Fig. 2b). Importantly, we found that the ischemic hepatic interventions alone (without follow-up cardiac intervention) did not cause hepatic cell damage, assessed by both liver histology and serum enzyme quantification. Thus, no visible cytoplasmic vacuolation, oedema, haemorrhage, or steatosis was found in liver sections (Fig. 3A). Furthermore, there was no difference in enzyme activity between RLIPC-treated and non-treated rats, as indicated by equal serum concentrations of AST, ALT, CK-MB, LDH, and α-HBDH (Fig. 3B).

### RLIPC induces cardioprotection against post-ischemic ventricular arrhythmias in STZ-induced diabetic rats

**Fig. 4:**
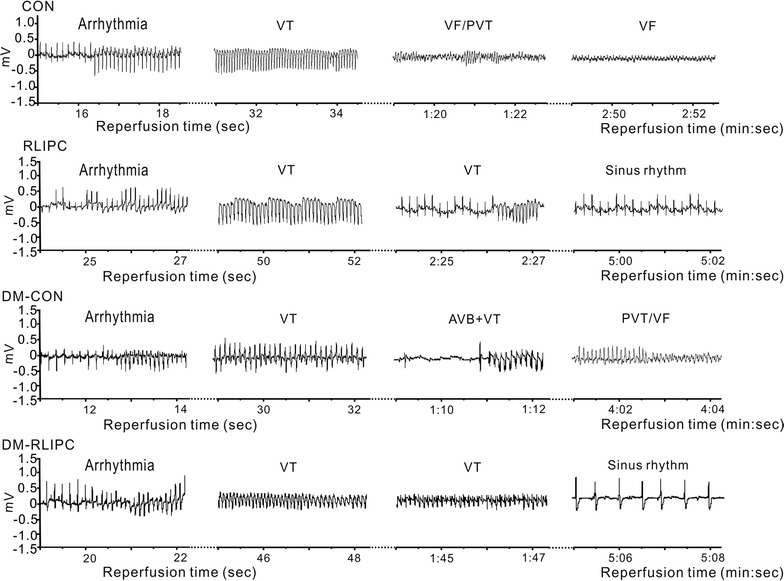
Representative effects of RLIPC on cardiac IRI-induced arrhythmias in diabetic and non-diabetic rats. Typical surface ECGs measured during the cardiac reperfusion period in diabetic and non-diabetic rats with or without RLIPC treatment. *CON* control, *DM* STZ-induced diabetes, *RLIPC* remote liver ischemia preconditioning, *VT* ventricular tachycardia, *PVT* polymorphic VT, *VF* ventricular fibrillation, *AVB* AV block

**Fig. 5:**
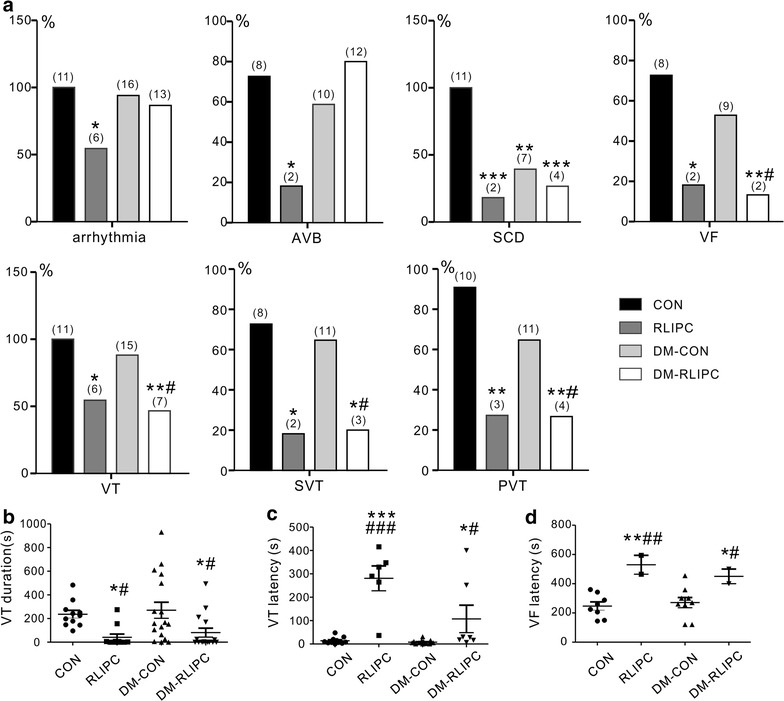
RLIPC confers differential cardioprotection against post-cardiac IRI arrhythmias and SCD in diabetic versus non-diabetic STZ-diabetic rats. **a** Quantification of arrhythmias during myocardial IRI in diabetic or nondiabetic rats with or without RLIPC (*n* = 11–17). Numbers of animals per category are indicated in *parentheses*. *AVB* AV block, *SCD* sudden cardiac death, *VF* ventricular fibrillation, *VT* ventricular tachycardia, *SVT* sustained ventricular tachycardia, *PVT* polymorphic VT, *CON* control, *RLIPC* remote liver ischemia conditioning, *DM* STZ-induced diabetes.*P < 0.05, **P < 0.01, ***P < *0.001* vs. CON, ^#^ *P* < 0.05 vs. DM-CON. **b** Mean VT durations for non-diabetic or STZ-induced diabetic rats with or without RLIPC (*n* = 11–17). Rats without VT were indicated as 0 s duration. *P < *0.05*, vs. CON, ^#^ *P* < *0.0*5 vs. DM-CON. **c**, **d** Latency to first run of **c** VT or **d** VF after the onset of reperfusion in non-diabetic or STZ-induced diabetic rats with or without RLIPC (*n* = 11–17). *P < *0.05*, **P < *0.01* and ***P < *0.001* vs. CON, ^#^ *P* < *0.05*, ^##^ *P* < *0.01* and ^###^ *P* < *0.001* vs. DM-CON

Rats in all experimental groups (but not sham-operated) underwent 5 min of left coronary artery ischemia followed by 20 min of reperfusion. Representative ECG tracings from healthy and diabetic rats with or without RLIPC treatment are presented in Fig. 4, and quantification shown in Fig. 5. The ratio of AAR size to the left ventricular size was similar among groups (data not shown). Classes of post-IRI arrhythmias that we observed included ventricular tachycardia (VT), sustained ventricular tachycardia (SVT), polymorphic ventricular tachycardia (PVT), ventricular fibrillation (VF), or atrioventricular block (AVB). RLIPC reduced the overall IRI-induced all-class arrhythmia incidence by almost half in non-diabetic rats (P = 0.035), but did not alter overall all-class arrhythmia incidence in diabetic rats. RLIPC was highly effective in reducing incidence of SCD (which resulted from VF or severe AVB post-IRI) in non-diabetic rats (2 out of 11, or 18%, compared to 11 out of 11, 100%, in control rats; P = 0.0002). Interestingly, SCD incidence was lower in control diabetic rats than in control non-diabetic rats (41%, 7/17, in DM-CON group, P = 0.0016), and was further reduced (to 27%, 4/15) in the DM-RLIPC group (Fig. 5a).

The fraction of nondiabetic rats exhibiting AVB was fourfold less in the RLIPC group (2/11) than in the control group (8/11) (P = 0.03), whereas RLIPC treatment failed to prevent AVB in diabetic rats, which was observed in 12/15 DM-RLPIC rats compared to 10/17 DM-CON rats. In contrast, the incidence of VT, or sustained (>1 min) VT (SVT),was high in both nondiabetic (11/11, 100%) and diabetic (15/17, 88%) control groups, but there was a striking protective effect of RLIPC against VT in RLIPC (6/11, P = 0.035) and DM-RLIPC (7/15, P = 0.0074) rats, and a decreased incidence of SVT from 73% (CON) to 18% in RLIPC (P = 0.03) or 20% in DM-RLIPC groups (P = 0.0149). Furthermore, 10 out of 11 (91%) healthy and 11/17 (65%) diabetic control rats who had VT degenerated into PVT, which is associated with VF or SCD in humans [17], while RLIPC was highly protective against PVT both in nondiabetic (incidence of 3/11, 27%, P = 0.0075) and diabetic (incidence of 4/15, 27%, P = 0.0017) rats. Furthermore, VF occurred in 73% (8/11) of control rats and 9/17 diabetic rats but its incidence was reduced to 18% in RLIPC-treated nondiabetic rats (P = 0.03) or 13% in RLIPC-treated diabetic rats (2/15, P = 0.004, by Fisher exact test) (Fig. 5a).

Control rats with (270.2 ± 67.8 s) or without (236.6 ± 32.8 s) STZ treatment had markedly longer mean VT duration throughout the reperfusion period than did RLIPC-treated non-diabetic (41.2 ± 27.3 s) or diabetic (80.9 ± 38.0 s) rats (P = 0.0045) (Fig. 5b). Although rats started to exhibit VT in the early phase of reperfusion (within 5 min post I/R), RLIPC successfully postponed the latency to the first run of VT after reperfusion, from 13.9 ± 4.3 s in the CON group, or 7.7 ± 2.1 s in the DM-CON group, to 280.8 ± 53.2 s in the RLIPC, or 107.3 ± 58.8 s in the DM-RLIPC group (P < 0.0001) (Fig. 5c). Notably, RLIPC also significantly delayed the latency to VF in both non-diabetic and diabetic rats (P = 0.0031) (Fig. 5d). Taken together, these results suggested that RLIPC exerts anti-arrhythmic effects post IRI in both non-diabetic and diabetic rats, albeit with some clear differences depending on arrhythmia type.

### Plasma glucose levels during reperfusion injury

**Fig. 6:**
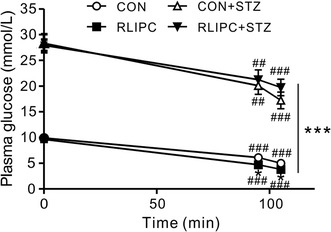
Plasma glucose levels in control and RLIPC diabetic and non-diabetic rats. Mean blood glucose measured in healthy and diabetic rats with or without RLIPC before and after myocardial ischemia followed by 20 min of reperfusion (*n* = 6–9). *P < *0.05*, ***P < *0.001* vs. CON. ^##^ *P* < *0.01*, ^###^ *P* < *0.001* vs. baseline, respectively

Diabetic rats exhibited higher plasma glucose levels than their non-diabetic counterparts throughout the experimental protocol, with the ratio increasing during reperfusion (as plasma glucose concentration dropped proportionately more in the non-diabetic groups than in diabetics). IRI resulted in a significant decrease of plasma glucose levels as compared to corresponding baseline parameters during cardiac reperfusion in each group (P < 0.01 or P < 0.001). While mean plasma glucose levels were lower in RLIPC-treated non-diabetic rats than in control rats during reperfusion (P < 0.05), no statistically significant differences in mean plasma glucose levels were detected between STZ-induced diabetic rats in response to RLIPC treatment, during a 20-min reperfusion period after left coronary artery ligation (Fig. 6).

### Analysis of RISK pathway protein phosphorylation

**Fig. 7:**
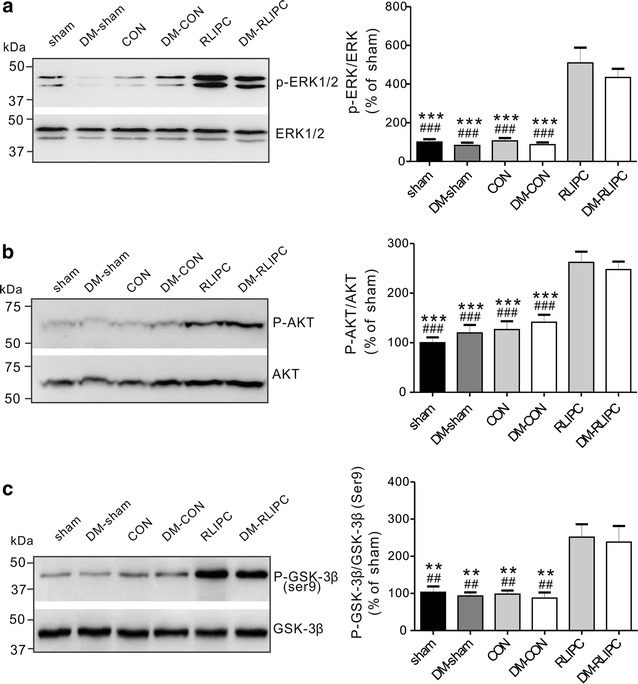
RLIPC induces similar ventricular RISK pathway protein phosphorylation in non-diabetic and diabetic rats. **a**–**c** (*Left*) Representative western blots and (*right*) band quantified ratios of phosphorylated versus total ventricular ERK (**a**), AKT (**b**) and GSK-3β (**c**) protein in sham, non-diabetic untreated (CON), non-diabetic RLIPC-treated (RLIPC), diabetic sham (DM-sham), diabetic untreated (DM-CON) and diabetic RLIPC-treated (DM-RLIPC) hearts post cardiac IRI. All band densities were normalized to sham (*n* = 5–7 per group). **P < 0.01, ***P < 0.001 vs. RLIPC; ^##^ *P* < 0.01, ^###^ *P* < 0.001 vs. DM-RLIPC

To further understand the RLIPC-induced cardioprotective effect in diabetic rat hearts, we investigated whether or not RLIPC altered the phosphorylation levels of proteins in the reperfusion injury salvage kinase (RISK) pathway, specifically ERK1/2, AKT, and GSK-3β. We did not find differences in total ERK1/2, AKT, and GSK-3β levels in any of the experimental groups, and levels of phosphorylated forms were thus normalized to total protein levels of each protein. Compared to control hearts, RLIPC dramatically and equally increased phosphorylation of post-cardiac reperfusion ventricular ERK1/2 (P < 0.001), AKT (P < 0.001) and GSK-3β (Ser9) (P = 0.01) in both diabetic and non-diabetic rat hearts (Fig. 7a–c).

## Discussion

### DM and cardiovascular disease

DM is a burgeoning threat to global human health. Aside from the well-known metabolic problems caused by DM, it increases the risk of SCD [3–5], which is already a major global cause of reduced quality of life and increased mortality [7]. Long-term, DM predisposes to coronary artery disease, itself a prominent cause of early death because it predisposes to heart attacks (distinct from SCD) in which plaque rupture leads to loss of blood flow to the heart. In addition, because DM is associated with increased incidence of myocardial ischemia, it generates a substrate for SCD. T1DM, arising from pancreatic β-cell destruction, is mechanistically distinct from the more common T2DM, which comprises both insulin resistance and impaired insulin secretion. However, T1DM and T2DM are each associated with abnormally elevated blood glucose concentration, and with similarly elevated cardiovascular mortality [18, 19]. Interestingly, in a comparison of nondiabetic, T1DM, and T2DM individuals in a Finnish population, hyperglycemia had a greater detrimental effect on risk of cardiovascular disease-related mortality in T1DM compared to T2DM individuals [18]. There is still much to learn about the specific aspects of DM that predispose to SCD, a correlation that holds even after adjusting for other cardiovascular risk factors caused by SCD [3, 9, 20, 21]. In the present study, we used an acute, streptozotocin-induced model of DM. This model replicates pancreatic aspects of T1DM (β-cell destruction), and rapidly achieves the hyperglycemia associated with both T1DM and T2DM, but does not incorporate the long-term vascular, metabolic, and other changes that also impact DM-related cardiovascular disease. As such, our study is most likely to shed light on effects upon arrhythmogenesis of hyperglycemia, and/or loss of control of blood glucose in general, rather than other, chronic aspects of DM.

### DM and remote ischemic preconditioning

RIPC, using transient ischemia/reperfusion as a stimuli in remote organs or limbs, has been indicated as an attractive strategy in clinical settings [22], and is cardioprotective in children undergoing open-heart surgery [23]. In patients undergoing coronary artery bypass graft (CABG) surgery, RIPC reduced incidence of POAF, preserved mitochondrial function and prevented MicroRNA upregulation in human right atrium [24]. However, acute hyperglycemia, induced by glucose infusion, was previously found to abolish RIPC-induced cardioprotection with respect to infarct size following 120 min of reperfusion. This effect was suggested to occur via an acute hyperglycemia-induced increase in nitrative stress and mTOR pathway [25]. Although we previously found that RLIPC exerts strong cardioprotective effects against arrhythmia and infarction, the effects of RLIPC on cardiac arrhythmia in subjects with diabetes mellitus, especially T1DM, are incompletely understood.

Here, to begin to address this, we imposed acute DM in rats by a single STZ treatment, ensuring that DM would be present 1 week later in the absence of other cardiovascular risk factors, and then we examined both the IRI-induced arrhythmias following an imposed ischemic event (coronary artery ligation), and the effect of DM on the therapeutic efficacy of RLIPC, an intervention we previously showed to be highly effective in reducing IRI arrhythmogenesis and mortality [12, 13]. Our new findings can be summarized as follows. The post-IRI incidence of all-class arrhythmia, AVB, VF, VT, SVT and PVT was similar for non-diabetic and diabetic rats, as were the mean VT duration and latencies to first VT and first VF. Unexpectedly, however, the incidence of SCD was lower in control diabetic than control non-diabetic rats. With respect to therapeutic efficacy, RLIPC was equally effective, in diabetic and nondiabetic rats, at reducing incidence of VT, SVT, PVT, and VF, reducing VT duration, and increasing latency to first VF. In contrast, AVB incidence (and, accordingly, all-class arrhythmia incidence) was effectively reduced by RLIPC in nondiabetic but not diabetic rats. SCD incidence was already lower in diabetics than nondiabetics, but RLIPC was relatively ineffective at reducing it further in diabetics, whereas in nondiabetics RLIPC was highly effective in this regard. Finally, latency to first VT in diabetic RLIPC rats was double that in nondiabetics. Interestingly, using a different protocol of remote limb postconditioning (RIPostC), Han and colleagues failed to find positive effects of remote conditioning in DM mice [26]. The discrepancy may result from differences in the treatment protocol, the animal disease model and the animal species, and emphasizes the importance of not assuming generality of ischemic conditioning effects across species or protocols.

### Resistance of AVB to RLIPC

The resistance to RLIPC of AVB in diabetic rats is of particular interest given that high-degree AVB has been shown in multiple human studies to occur more frequently in diabetics than nondiabetics [27]. We saw no DM-dependent difference in AVB incidence in control groups, but rather a lack of therapeutic response in diabetic rats. As we imposed an ischemic event (coronary artery ligation) one could argue that we eliminated the DM-dependent factor responsible for increased AVB incidence, i.e., myocardial ischemia, but studies of human populations have suggested that DM predisposes to AVB even after adjusting for other DM-related cardiovascular risk factors including coronary artery disease, heart failure and hyperlipidemia [28, 29]. One potential reason underlying the increased prevalence of high-degree AVB in human diabetics is diabetic microangiopathy, and as this is a feature of long-term DM [30], it would not be expected to be present in the rats in our acute study, so could be one of the factors explaining why we did not see increased AVB at baseline in the diabetic group. Of more significance is the lack of response to RLIPC in the diabetic group. This cannot be explained by differential induction of RISK pathway protein phosphorylation, as this was similar between diabetic and nondiabetic rats (Fig. 7). Phosphorylation of RISK pathway proteins, and specifically inhibition of GSK-3β (either by pharmacological inhibitors or preconditioning-induced phosphorylation at serine 9, which results in inhibition of GSK-3β, as we found here in response to RLIPC) is established to be cardioprotective in the context of IRI, in terms of reducing infarct size, reducing cell death, and reducing incidence of ventricular arrhythmias and SCD [12, 15, 31–33]. To our knowledge, this is the first report showing that induction of GSK-3β serine 9 phosphorylation is DM-specifically ineffective against IRI-induced AVB.

One interesting possible mechanism to consider for the resistance of AVB to RLIPC in diabetic rats is plasma glucose levels, which, as expected, remained very high during and after IRI regardless of RLIPC (>20 mmol/L in DM-RLIPC rats) (Fig. 6). It was recently found that elevated admission glycaemia (>10.05 mmol/L) in human patients with ST-segment elevation myocardial infarction was independently associated with increased risk of high-grade AVB [34]. Ischemia or infarction are common causes of AVB, but it can also be produced by neurally mediated vagal reflex [35]. Autonomic perturbation and hyperglycemia were previously found to be interdependent in human non-diabetic stroke patients [36], and in a fructose model of glucose intolerance in mice, cardiac sympatho-vagal balance was altered within 15 days of fructose administration, preceding metabolic dysfunction [37]. Thus, it is possible that AVB was resistant to RLIPC in the current study because of overarching effects of vagal tone, a possibility to be investigated in future work.

### SCD, RLIPC and DM

Similarly, the lower incidence of SCD in control diabetic rats compared to control nondiabetic rats cannot be explained by differences in protein phosphorylation within the RISK pathway as these were not observed. It is possible that other cardioprotective pathways are induced by the acute STZ-induced diabetes, and that these lower risk of SCD despite equivalent IRI-induced arrhythmogenesis in diabetic and nondiabetic control (non-RLIPC-treated) rats. This would not tally with the increased risk of SCD seen in human DM compared to euglycemics, but it is important to note that under our experimental conditions, DM is acutely induced, and also that the rats in our study are under anesthesia and artificial ventilation, unlike most real-life scenarios involving human SCD. We recently found that targeted deletion in mice of the *Kcne2* potassium channel β subunit predisposes to SCD in non-fasted animals, and AVB in fasted animals, following IRI induced by a high ligation of the coronary artery [16]. In contrast, *Kcne2* deletion was cardioprotective, reducing infarct size and cardiac tissue damage, in mice following a lower ligation of the coronary artery designed to promote infarct but a lesser degree of early arrhythmogenesis [15]. *Kcne2* is ubiquitously expressed, in the heart but also pancreatic islet cells, gastric parietal cells, the thyroid and other epithelia, and its deletion also causes diabetes, atherosclerosis and fatty liver in mice [16, 38–42]; *Kcne2* polymorphisms also influence risk if coronary artery disease in human populations [43, 44]. Thus, even monogenic arrhythmia syndromes can exhibit complex pathogenesis and opposite outcomes depending on the nature of the cardiac ischemic insult, for example. We may be observing similar complexity herein, with respect to the unexpectedly low incidence of SCD in our untreated diabetic rats.

Interestingly, glucagon-like peptide 1 (GLP-1), the primary actions of which are to stimulate insulin secretion and to inhibit glucagon secretion (to dampen postprandial glucose spikes) [45], protects against ischemia reperfusion injury in human heart via a mechanism that is thought to be independent of mitochondrial K_ATP_ channels and therefore independent of RISK/SAFE pathway-dependent activation of mitochondrial K_ATP_ channels [46]. Thus, GLP-1 is thought to be cardioprotective via alternative mechanisms to those important in ischemic preconditioning, and to the RISK pathway which we show herein to be induced in RLIPC. It will be of interest in the future to examine the effects of GLP-1 on SCD and AVB in the streptozotocin-induced diabetic rat model.

## Limitations

We acknowledge several limitations of this study. First, we found that although IRI-induced arrhythmia was similar in nondiabetic and diabetic rats, the latter exhibited a lower incidence of SCD, contrasting with human population data. It is important to stress that our study utilized an experimentally induced animal model of hyperglycemia (streptozotocin-induced T1DM) rather than a longer-term model that would incorporate in addition the chronic effects of T1DM or T2DM, primarily vascular complications, known to be highly influential in DM-related cardiovascular disease and mortality. These chronic changes take years to develop in humans and are clearly not reflected in our acute model. Second, we used only one RLIPC treatment strategy; thus, the possible influence of other RLIPC protocols on, e.g., AVB, cannot be absolutely excluded. Third, we used a rat model, and as mentioned above, the responsiveness to arrhythmia stimuli may vary differently between species. Fourth, the primary goal of our study was to ascertain whether RLIPC is beneficial in the context of post IRI cardiac arrhythmias in hyperglycemic rats. We did not evaluate serum electrolytes during this protocol and therefore the possibility remains that differential IRI-induced electrolyte imbalances between nondiabetic and DM rats contributed to differential effects of IRI and/or RLIPC.

## Conclusions

In summary, acute STZ-induced DM did not substantively alter IRI arrhythmia induction in rats, but it unexpectedly lowered SCD incidence. While the therapeutic efficacy of RLIPC against ventricular tachyarrhythmias, and its ability to induce ventricular RISK pathway protein phosphorylation, were not diminished by DM, RLIPC was ineffective against IRI-induced AVB, a malignant arrhythmia with increased incidence in human diabetics. Future studies could pursue possible molecular remodeling in the AV node during DM and/or IRI with the goal of identifying potential therapeutic avenues distinct from those involved in pathogenesis, and cardioprotection against, IRI-induced ventricular tachyarrhythmia.

## Abbreviations

ALT: alanine aminotransferase
AST: aspartate aminotransferase
AVB: atrioventricular block
AAR: area at risk
CABG: coronary artery bypass graft surgery
CK: creatine kinase
DM: diabetes mellitus
ERK: extracellular signal-regulated kinase
GLP-1: glucagon-like peptide-1
GSK-3β: glycogen synthase kinase-3β
α-HBDH: α-hydroxybutyrate dehydrogenase
IRI: ischemia reperfusion injury
LDH: lactate dehydrogenase
mTOR: mechanistic target of rapamycin
NSCD: non-sudden cardiac death
PVT: polymorphic VT
POAF: post-operative atrial fibrillation
RIPC: remote ischemia preconditioning
RLIPC: remote liver ischemia preconditioning
RISK: reperfusion injury salvage kinase
SCA: sudden cardiac arrest
SCD: sudden cardiac death
STZ: streptozotocin
T1DM: type 1 diabetes mellitus
T2DM: type 2 diabetes mellitus
VF: ventricular fibrillation
VT: ventricular tachycardia
